# Predictive role of pretreatment skeletal muscle mass index for long-term survival of bladder cancer patients: A meta-analysis

**DOI:** 10.1371/journal.pone.0288077

**Published:** 2023-06-30

**Authors:** Qian Yuan, Jianrong Hu, Feng Yuan, Jingjing An

**Affiliations:** 1 Department of Anesthesiology, West China Hospital, Sichuan University, Chengdu, China; 2 West China School of Nursing, Sichuan University, Chengdu, Sichuan, China; Mater Olbia Hospital, ITALY

## Abstract

**Purpose:**

To identify the predictive role of pretreatment skeletal muscle mass index (SMI) for long-term survival of bladder cancer patients.

**Methods:**

Several databases were searched for studies investigating the relationship between pretreatment SMI and prognosis in bladder cancer. The overall survival (OS) and cancer-specific survival (CSS) were defined as primary and secondary outcomes, respectively. Hazard ratios (HRs) and 95% confidence intervals (CIs) were combined.

**Results:**

Nine studies involving 1476 cases were included. The results demonstrated that a lower pretreatment SMI was significantly related to poorer OS (HR = 1.56, 95% CI: 1.33–1.82, P<0.001) and subgroup analysis based on thresholds of SMI revealed similar results. Besides, pretreatment SMI was also obviously related to CSS (HR = 1.75, 95% CI: 1.36–2.25, P<0.001).

**Conclusion:**

Lower pretreatment SMI was associated with worse long-term survival of bladder cancer patients.

## Introduction

Bladder cancer remains one of the most common urinary malignancies and mainly occurs in the elderly patients [1]. According to the latest cancer data, there was 570,000 new cases in 2020 all over the world, with the tenth morbidity [2]. For male patients, 440,000 bladder cancer cases occur with the sixth morbidity and 160,000 patients died with the ninth mortality among all cancers in 2020 [2]. Besides, the incidence tends to increase gradually, which causes a certain tumor burden to the society. Up to now, the prognosis of bladder cancer is still poor despite advances in surgical technologies and chemotherapy [3, 4].

Increasing evidence has demonstrated that tumor progression and prognosis depend not only on the biological aggressiveness of the tumor but also on the host’s response to the tumor. Host factors such as the nutritional status and local or systemic inflammation response are also important indicators of clinical treatment [5]. Systemic inflammation response index (SII) has been verified to be significantly associated with treatment response and survival of bladder cancer patients [6, 7]. Loss of weight and body mass index (BMI) are usually applied to evaluate the nutritional status and cachexia in cancer patients, but these indexes only reflect the total body composition and do not distinguish the proportion and change of fat and muscle mass. Actually, the muscle mass is significantly related to the overall body condition and nutritional status of cancer patients [8, 9].

Many studies have manifested that sarcopenia could reflect potential malnutrition and weakness caused by cancers and skeletal muscle mass index (SMI) is the most authoritative indicator to evaluate the presence or absence of sarcopenia in cancer patients [10, 11]. SMI is calculated by dividing the total area of all skeletal muscles, including the psoas major muscle, erector spinae muscle, quadratus lumborum muscle, transverse muscle of abdomen, obliquus externus abdominis and obliquus internus abdominis, in the third lumbar level of CT images by the square of height [12]. Up to now, the association of pretreatment SMI with long-term survival has been verified by meta-analyses in several types of cancers like the lung cancer [12, 13]. However, the prognostic value of pretreatment SMI in bladder cancer remains unclear now.

Therefore, the aim of this meta-analysis was to identify predictive role of pretreatment SMI for long-term survival of bladder cancer patients.

## Materials and methods

This meta-analysis was performed according to the Preferred Reporting Items for Systematic Reviews and Meta-Analysis (PRISMA 2020) checklist [14]. The detailed checklist information was presented in the S1 File.

### Literature search

The PubMed, EMBASE, WOS and CNKI database were searched up to September 21, 2022. Terms used during the literature search are as follows: skeletal muscle mass index, SMI, bladder, tumor, cancer, neoplasm, carcinoma, survival, prognostic and prognosis. Search strategy was as follows: (skeletal muscle mass index OR SMI) AND bladder AND (tumor OR cancer OR neoplasm OR carcinoma) AND (survival OR prognostic OR prognosis). Besides, the free texts and MeSH terms were used.

### Inclusion criteria

The inclusion criteria included: 1) patients were diagnosed with primary bladder cancer; 2) SMI was calculated according to the CT images of the third lumbar vertebra as previously reported [15]; 3) the SMI values were obtained before anti-tumor therapy such as the surgery and chemoradiotherapy; 4) patients were divided into two groups according to values of SMI and long-term survival representing as the overall survival (OS) and cancer-specific survival (CSS) were compared; 5) hazard ratios (HRs) and 95% confidence intervals (CIs) were provided in the articles.

### Exclusion criteria

The exclusion criteria included: 1) letters, editorials, case reports, reviews or animal trials; 2) duplicated or overlapped data; 3) insufficient information for methodological quality assessment.

### Data extraction

Data were collected from included studies: the name of first author, publication year, country, sample size, tumor-node-metastasis (TNM) stage, treatment (surgery or non-surgery), cutoff value of SMI, endpoint, HR and 95% CI.

### Methodological quality assessment

Methodological quality was evaluated according to Newcastle-Ottawa Scale (NOS) score due to the retrospective nature of study design [16]. Studies with a NOS score ≥6 were regarded as high-quality studies.

### Statistical analysis

Statistical analysis was conducted by STATA 15.0 software. HRs with 95% CIs were combined to assess the relationship between pretreatment SMI and prognosis of bladder cancer patients. The heterogeneity among included studies was evaluated by I^2^ statistics and Q test. When significant heterogeneity was observed representing as I^2^ > 50% and (or) P < 0.1, the random-effects model was applied; otherwise, the fix-effects model was applied. The sensitivity analysis was performed to evaluate stability of results. Furthermore, Begg’s funnel plot and Egger’s test were conducted to detect publication bias [17, 18].

## Results

### Literature search

**Fig 1:**
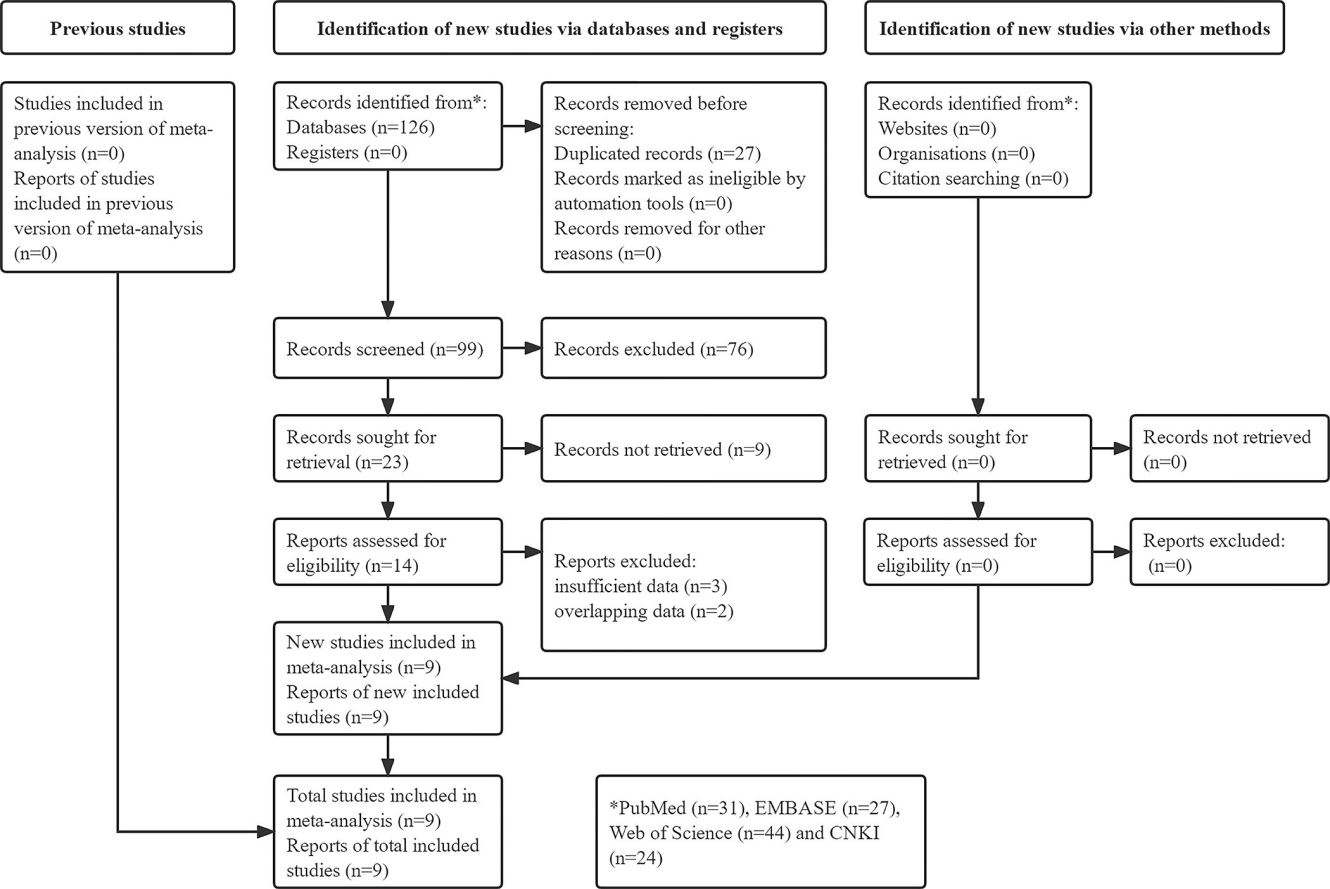
Prisma flow diagram of this meta-analysis.

A total of 126 records were identified from databases and 27 duplicated records were removed. Eventually, nine studies were included [19–27]. The detailed selection process was shown in the **Fig 1**.

### Basic characteristics of included studies

Among nine retrospective included studies, 1476 patients were enrolled and the sample size ranged from 80 to 500 [19–27]. Among four of included studies the cutoff values of SMI, 55cm^2^/m^2^ for male and 39cm^2^/m^2^ for female, were applied [19, 21, 24, 27]. In the other five studies, the cutoff values of SMI, 43/53cm^2^/m^2^ for male and 39cm^2^/m^2^ for female, were applied and the cutoff value of SMI for male patients was adjusted by the body mass index (BMI), SMI <43cm^2^/m^2^ for patients with BMI<25kg/m^2^ and <53cm^2^/m^2^ for patients with BMI≥25 kg/m^2^ [20, 22, 23, 25, 26]. All included studies were with high-quality with a NOS score ≥6. Specific information was displayed in **Table 1**.

**Table 1:** Basic characteristics of included studies.

Author	Year	Country	Sample size	TNM stage	Treatment	Cutoff value	Endpoint	NOS
Psutka [19]	2014	USA	205	Mixed	Surgery	male: 55cm^2^/m^2^, female: 39cm^2^/m^2^	OS, CSS	7
Miyake [20]	2017	Japan	89	NR	Surgery	male: 43/53cm^2^/m^2^, female: 41cm^2^/m^2^	OS, CSS	7
Abe [21]	2018	Japan	87	NR	Chemotherapy/ chemotherapy plus surgery	male: 55cm^2^/m^2^, female: 39cm^2^/m^2^	OS	6
Mayr [22]	2018	Netherlands	500	Mixed	Surgery	male: 43/53cm^2^/m^2^, female: 41cm^2^/m^2^	OS, CSS	7
Ha [23]	2019	Republic of Korea	80	Mixed	Surgery	male: 43/53cm^2^/m^2^, female: 41cm^2^/m^2^	OS	7
Lyon [24]	2019	USA	183	Mixed	Surgery	male: 55cm^2^/m^2^, female: 39cm^2^/m^2^	OS, CSS	7
Stangl [25]	2019	Austria	94	Mixed	Radiotherapy	male: 43/53cm^2^/m^2^, female: 41cm^2^/m^2^	OS, CSS	6
Yuan [26]	2021	China	97	cT1-2	Surgery	male: 43/53cm^2^/m^2^, female: 41cm^2^/m^2^	OS	8
Almarzouq [27]	2022	Canada	141	Mixed	Radiotherapy plus chemotherapy	male: 55cm^2^/m^2^, female: 39cm^2^/m^2^	OS	6

NR: not reported; OS: overall survival; CSS: cancer-specific survival; NOS: Newcastle-Ottawa Scale.

### The predictive role of pretreatment SMI for OS in bladder cancer

**Fig 2:**
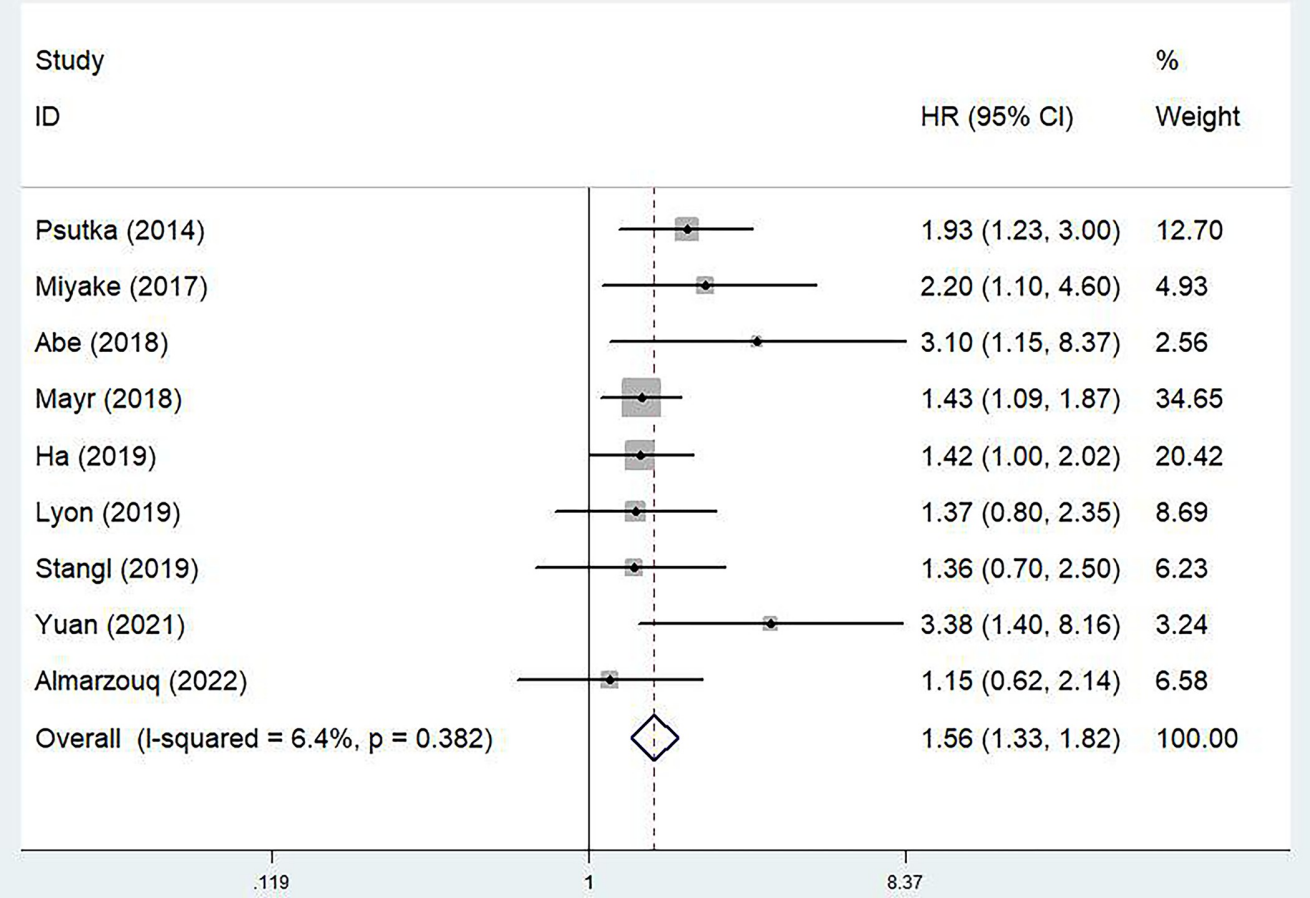
The association between pretreatment skeletal muscle mass index and overall survival of bladder cancer patients.

All included studies explored predictive role of pretreatment SMI for OS [19–27]. Pooled results indicated that a lower pretreatment SMI was significantly associated with poor OS in bladder cancer (HR = 1.56, 95% CI: 1.33–1.82, P<0.001; I^2^ = 6.4%, P = 0.382) **(Fig 2)**. Subgroup analysis stratified by thresholds of SMI showed similar results (non-adjusted SMI: HR = 1.63, 95% CI: 1.22–2.17, P = 0.001; BMI-adjusted: HR = 1.52, 95% CI: 1.26–1.84, P<0.001) **(Table 2)**.

**Table 2:** Results of meta-analysis.

	No. of studies	HR	95% CI	P value	I^2^ (%)	P value
Overall survival	9 [19–27]	1.56	1.33–1.82	<0.001	6.4	0.382
Cutoff value of SMI						
Non-adjusted	4 [19, 21, 24, 27]	1.63	1.22–2.17	0.001	20.7	0.286
BMI-adjusted	5 [20, 22, 23, 25, 26]	1.52	1.26–1.84	<0.001	13.4	0.329
Cancer-specific survival	5 [19, 20, 22, 24, 25]	1.75	1.36–2.25	<0.001	15.5	0.316

HR: hazard ratio; CI: confidence interval; SMI: skeletal muscle mass index.

### The predictive role of pretreatment SMI for CSS in bladder cancer

**Fig 3:**
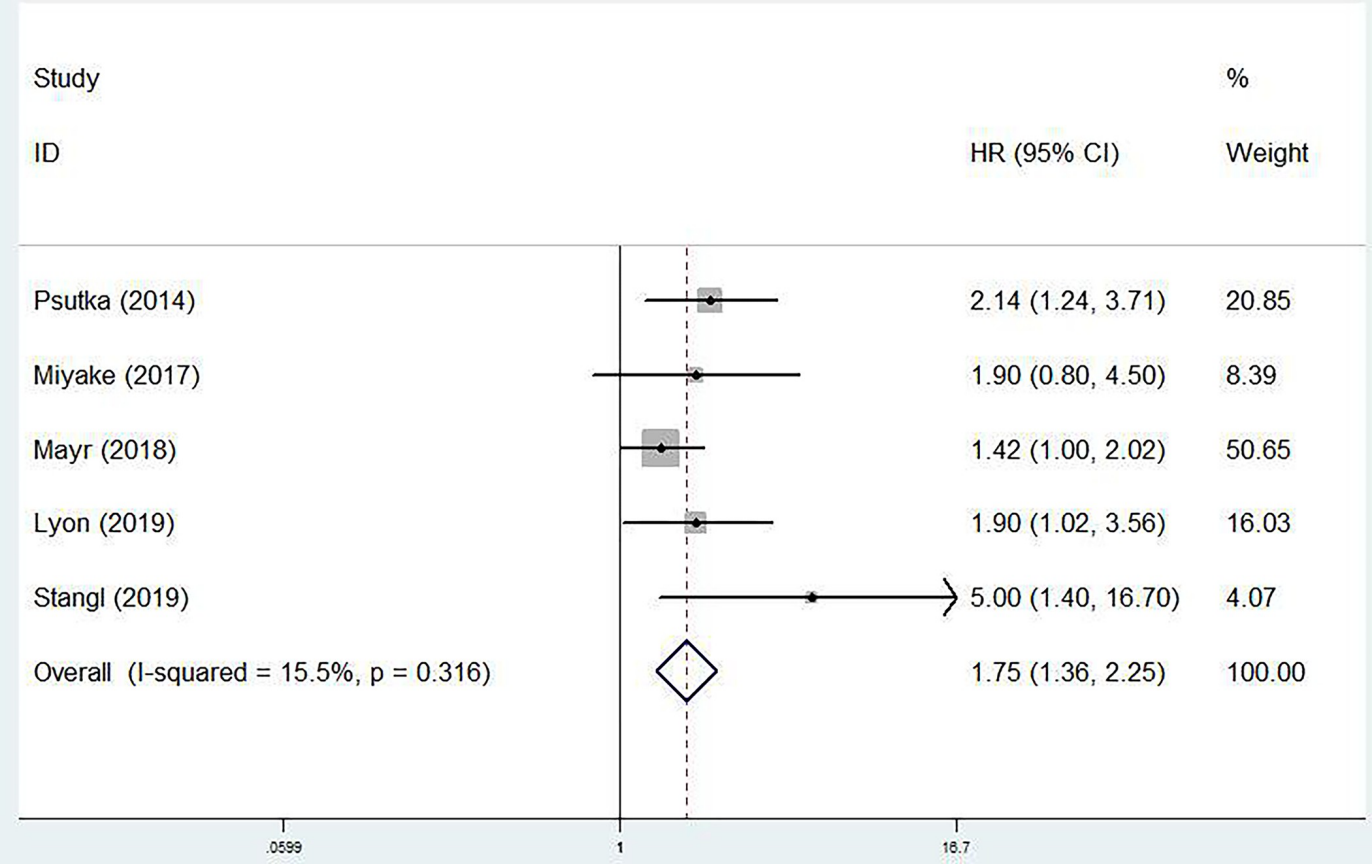
The association between pretreatment skeletal muscle mass index and cancer-specific survival of bladder cancer patients.

Five studies investigated predictive role of SMI for CSS of bladder cancer patients [19, 20, 22, 24, 25]. Pooled results revealed that lower pretreatment SMI was related to worse CSS (HR = 1.75, 95% CI: 1.36–2.25, P<0.001; I^2^ = 15.5%, P = 0.316) **(Fig 3)**.

### Sensitivity analysis and publication bias

**Fig 4:**
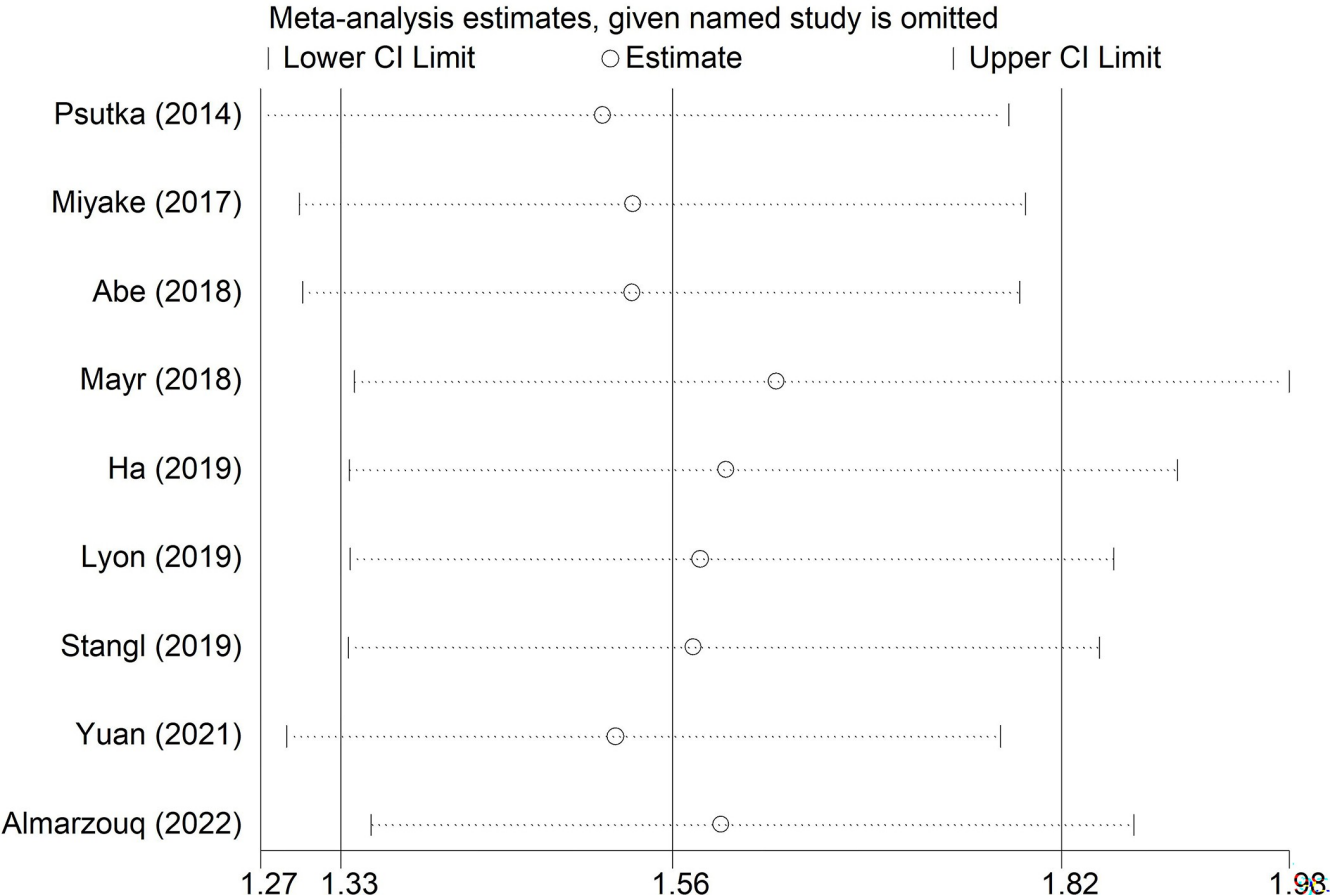
Sensitivity analysis about the association between pretreatment skeletal muscle mass index and overall survival of bladder cancer patients.

**Fig 5:**
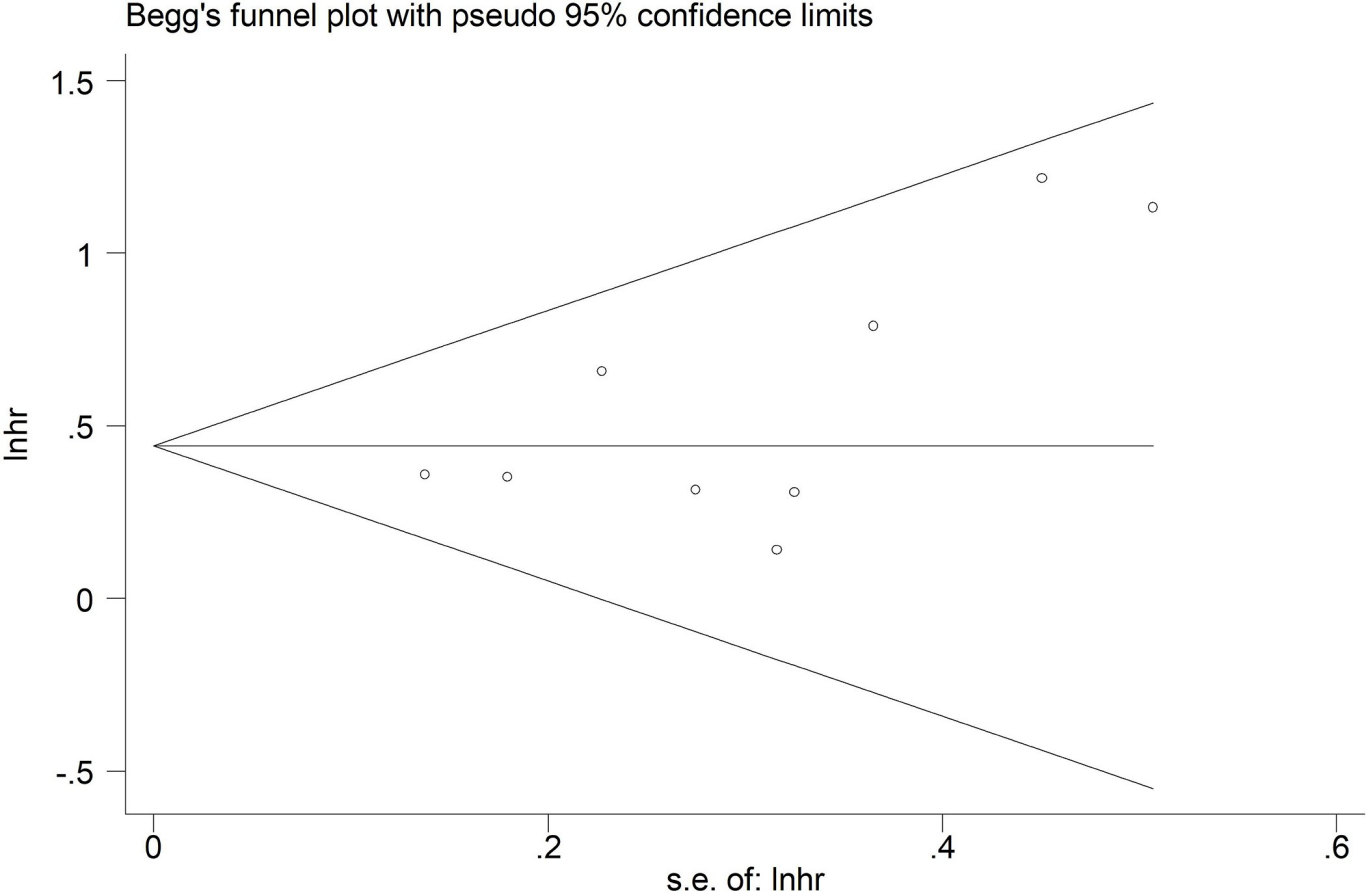
Begg’s funnel plot.

Sensitivity analysis for OS demonstrated that our results were stable and reliable **(Fig 4)**. Besides, symmetrical Begg’s funnel plot **(Fig 5)** and P = 0.096 of Egger’s test both indicated non-significant publication bias.

## Discussion

The current meta-analysis demonstrated that pretreatment SMI was associated with long-term survival in bladder cancer and lower pretreatment SMI predicted poorer OS and CSS. Therefore, pretreatment SMI might serve as a reliable prognostic indicator in bladder cancer. However, due the limitations existed in this meta-analysis like to the retrospective nature of included studies more prospective high-quality studies are still needed to verify our results.

Actually, the predictive role of SMI for survival in cancers has been verified. Pan et al. included 12 studies involving 3002 cases and demonstrated that a lower SMI was obviously related to poorer OS (HR = 1.23, P<0.001) [12]. The subgroup analysis based on treatment, stage and tumor type further manifested prognostic role of SMI in lung cancer and showed similar results [12]. Yao et al. enrolled 2441 patients from 17 studies and demonstrated that lower pretreatment SMI was associated with poorer OS (HR = 1.18, P<0.001) and disease-free survival (DFS) (HR = 1.78, P = 0.019) [13]. Subgroup analysis stratified by the treatment, tumor type and thresholds of SMI revealed similar findings [13]. Our meta-analysis was the first to determine predictive role of pretreatment SMI in bladder cancer and strongly verified that lower pretreatment SMI was related to worse prognosis.

SMI is the most common indicator assessing the presence or absence of sarcopenia. Initially, sarcopenia is regarded as a disease of old age characterized by degeneration of muscle tissue. However, increasing evidence indicated that a number of factors could cause sarcopenia such as the disuse, cachexia, malabsorption and also tumors [28]. Meanwhile, the occurrence and development of sarcopenia are closely related to the prognosis of cancer patients [13]. Sarcopenia includes physiological and pathological sarcopenia and the latter is caused by malignant or benign diseases. Tumor-associated sarcopenia is usually closely related to cachexia, representing as marked muscle mass loss and systemic chronic inflammation [29, 30]. The incidence rate of tumor-associated sarcopenia is about 50%-90% in untreated cancer patients [31]. Among patients with bladder cancer, the occurrence rate of sarcopenia is more than 50% [32]. Bladder cancer patients may experience malnutrition due to the impact of the tumor on the body’s metabolism and absorption, or due to adverse reactions during treatment, such as loss of appetite, nausea, and vomiting. Besides, bladder cancer patients with sarcopenia may have reduced tolerance to surgical and chemotherapeutic treatments, and the disruption of the body’s immune and metabolic functions may interfere with the normal response to these treatments [33]. In the past years, the association between sarcopenia and prognosis in cancers has been widely reported and revealed. For now, the predictive role of sarcopenia has been confirmed in several types of tumors including esophageal cancer, rectal cancer and hepatocellular carcinoma [34–37]. Therefore, our meta-analysis indirectly proved that the sarcopenia assessed by SMI before any anti-tumor treatment was a novel and reliable prognostic factor in bladder cancer.

In the current meta-analysis, we failed to conduct more analysis about clinical role of SMI in bladder cancer because of lack of original data and limited current evidence. There are still many fields worthy of further investigations. For example, our meta-analysis only identified the association between pretreatment SMI and long-term survival. However, whether the change of SMI during the anti-tumor treatment could predict survival and contribute to the therapy strategy remains unclear. Besides, the cutoff values of SMI are gender-specific and BMI is sometimes considered. It is not clear whether more parameters should be considered such as the age and tumor stage. Furthermore, skeletal muscle plays an essential role in the systemic inflammation response and a large number of evidences have shown that the status of systemic inflammation response is closely related to prognosis of cancer patients [38–40]. Thus, a combination of SMI and some inflammation indexes like the SII might be better in predicting the long-term survival of bladder cancer patients.

### Limitation of this study

Several limitations exist in this meta-analysis. First, all included studies are retrospective with relatively small sample sizes, which might cause some bias. Second, some clinicopathological parameters are unobtainable such as the TNM stage and age and we were unable to conduct more subgroup analysis based on these important indicators due to the lack of original data.

## Conclusion

Lower pretreatment SMI was associated with worse long-term survival of bladder cancer patients. However, more prospective high-quality studies are still needed to verify our results.

## Supporting information

S1 File PRISMA 2020 checklist for this meta-analysis. (DOCX)

## References

[1] Ferlay J, Colombet M, Soerjomataram I, Mathers C, Parkin DM, Piñeros M, et al. Estimating the global cancer incidence and mortality in 2018: GLOBOCAN sources and methods. International journal of cancer 2019, 144(8):1941–1953. doi: 10.1002/ijc.31937 30350310

[2] Sung H, Ferlay J, Siegel RL, Laversanne M, Soerjomataram I, Jemal A, et al. Global Cancer Statistics 2020: GLOBOCAN Estimates of Incidence and Mortality Worldwide for 36 Cancers in 185 Countries. CA: a cancer journal for clinicians 2021, 71(3):209–249. doi: 10.3322/caac.21660 33538338

[3] Xia C, Dong X, Li H, Cao M, Sun D, He S, et al. Cancer statistics in China and United States, 2022: profiles, trends, and determinants. Chinese medical journal 2022, 135(5):584–590. doi: 10.1097/CM9.0000000000002108 35143424 PMC8920425

[4] Kang MJ, Won YJ, Lee JJ, Jung KW, Kim HJ, Kong HJ, et al. Cancer Statistics in Korea: Incidence, Mortality, Survival, and Prevalence in 2019. Cancer research and treatment 2022, 54(2):330–344. doi: 10.4143/crt.2022.128 35313102 PMC9016309

[5] Fukushima H, Yokoyama M, Nakanishi Y, Tobisu K, Koga F. Sarcopenia as a prognostic biomarker of advanced urothelial carcinoma. PLoS One 2015, 10(1):e0115895. doi: 10.1371/journal.pone.0115895 25612215 PMC4303429

[6] Ye K, Xiao M, Li Z, He K, Wang J, Zhu L, et al. Preoperative systemic inflammation response index is an independent prognostic marker for BCG immunotherapy in patients with non-muscle-invasive bladder cancer. Cancer medicine 2022. 10.1002/cam4.5284 PMC9972176 36214475

[7] Ke ZB, Chen H, Chen JY, Cai H, Lin YZ, Sun XL, et al. Preoperative abdominal fat distribution and systemic immune inflammation were associated with response to intravesical Bacillus Calmette-Guerin immunotherapy in patients with non-muscle invasive bladder cancer. Clinical nutrition (Edinburgh, Scotland) 2021, 40(12):5792–5801. 34775222 10.1016/j.clnu.2021.10.019

[8] Tanaka K, Taoda A, Kashiwagi H. The associations between nutritional status, physical function and skeletal muscle mass of geriatric patients with colorectal cancer. Clinical nutrition ESPEN 2021, 41:318–324. doi: 10.1016/j.clnesp.2020.11.009 33487284

[9] Fang Z, Du F, Shang L, Liu J, Ren F, Liu Y, et al. CT assessment of preoperative nutritional status in gastric cancer: severe low skeletal muscle mass and obesity-related low skeletal muscle mass are unfavorable factors of postoperative complications. Expert review of gastroenterology & hepatology 2021, 15(3):317–324. 33063547 10.1080/17474124.2021.1836959

[10] Wang K, Long W, Sima X, Zhao Y, Xiao B, Gulizeba H, et al. Sarcopenia defined by skeletal muscle mass index at the third lumbar vertebra is a prognostic factor for extensive-stage small cell lung cancer patients: a retrospective study. Journal of thoracic disease 2022, 14(7):2645–2651. 35928624 10.21037/jtd-22-782 PMC9344420

[11] Kawakami R, Tanisawa K, Ito T, Usui C, Miyachi M, Torii S, et al. Fat-Free Mass Index as a Surrogate Marker of Appendicular Skeletal Muscle Mass Index for Low Muscle Mass Screening in Sarcopenia. Journal of the American Medical Directors Association 2022. 10.1016/j.jamda.2022.08.016 36179769

[12] Pan XL, Li HJ, Li Z, Li ZL. Prognostic value of computed tomography derived skeletal muscle mass index in lung cancer: A meta-analysis. World journal of clinical cases 2022, 10(20):6927–6935. 36051119 10.12998/wjcc.v10.i20.6927 PMC9297422

[13] Yao L, Wang L, Yin Y, Che G, Yang M. Prognostic Value of Pretreatment Skeletal Muscle Mass Index in Esophageal Cancer Patients: A Meta-Analysis. Nutrition and cancer 2022, 74(10):3592–3600. 35730425 10.1080/01635581.2022.2088814

[14] Salameh JP, Bossuyt PM, McGrath TA, Thombs BD, Hyde CJ, Macaskill P, et al. Preferred reporting items for systematic review and meta-analysis of diagnostic test accuracy studies (PRISMA-DTA): explanation, elaboration, and checklist. BMJ (Clinical research ed) 2020, 370:m2632. 10.1136/bmj.m2632 32816740

[15] Regnier P, De Luca V, Brunelle S, Sfumato P, Walz J, Rybikowski S, et al. Impact of sarcopenia status of muscle-invasive bladder cancer patients on kidney function after neoadjuvant chemotherapy. Minerva Urology and Nephrology 2021, 73(2):215–224. 32083413 10.23736/S2724-6051.20.03616-4

[16] Wang Y, Li J, Chang S, Dong Y, Che G. Risk and Influencing Factors for Subsequent Primary Lung Cancer After Treatment of Breast Cancer: A Systematic Review and Two Meta-Analyses Based on Four Million Cases. Journal of thoracic oncology: official publication of the International Association for the Study of Lung Cancer 2021, 16(11):1893–1908. 34256110 10.1016/j.jtho.2021.07.001

[17] Begg CB, Mazumdar M. Operating characteristics of a rank correlation test for publication bias. Biometrics 1994, 50(4):1088–1101. 7786990

[18] Egger M, Davey Smith G, Schneider M, Minder C. Bias in meta-analysis detected by a simple, graphical test. BMJ (Clinical research ed) 1997, 315(7109):629–634. 10.1136/bmj.315.7109.629 PMC2127453 9310563

[19] Psutka SP, Carrasco A, Schmit GD, Moynagh MR, Boorjian SA, Frank I, et al. Sarcopenia in patients with bladder cancer undergoing radical cystectomy: Impact on cancer-specific and all-cause mortality. Cancer 2014, 120(18):2910–2918. 24840856 10.1002/cncr.28798

[20] Miyake M, Morizawa Y, Hori S, Marugami N, Shimada K, Gotoh D, et al. Clinical impact of postoperative loss in psoas major muscle and nutrition index after radical cystectomy for patients with urothelial carcinoma of the bladder. BMC Cancer 2017, 17(1):237. 28359307 10.1186/s12885-017-3231-7 PMC5374611

[21] Abe H, Takei K, Uematsu T, Tokura Y, Suzuki I, Sakamoto K, et al. Significance of sarcopenia as a prognostic factor for metastatic urothelial carcinoma patients treated with systemic chemotherapy. International Journal of Clinical Oncology 2018, 23(2):338–346. 29098519 10.1007/s10147-017-1207-x

[22] Mayr R, Gierth M, Zeman F, Reiffen M, Seeger P, Wezel F, et al. Sarcopenia as a comorbidity-independent predictor of survival following radical cystectomy for bladder cancer. Journal of Cachexia Sarcopenia and Muscle 2018, 9(3):505–513. 29479839 10.1002/jcsm.12279 PMC5989852

[23] Ha Y-S, Kim SW, Kwon TG, Chung SK, Yoo ES. Decrease in skeletal muscle index one year after radical cystectomy as a prognostic indicator in patients with urothelial bladder cancer. International Braz J Urol 2019, 45(4):686–694. 30901172 10.1590/S1677-5538.IBJU.2018.0530 PMC6837591

[24] Lyon TD, Frank I, Takahashi N, Boorjian SA, Moynagh MR, Shah PH, et al. Sarcopenia and Response to Neoadjuvant Chemotherapy for Muscle-Invasive Bladder Cancer. Clinical Genitourinary Cancer 2019, 17(3). 10.1016/j.clgc.2019.03.007 31060857

[25] Stangl-Kremser J, D’Andrea D, Vartolomei M, Abufaraj M, Goldner G, Baltzer P, et al. Prognostic value of nutritional indices and body composition parameters including sarcopenia in patients treated with radiotherapy for urothelial carcinoma of the bladder. Urologic oncology 2019, 37(6):372–379. 30578161 10.1016/j.urolonc.2018.11.001

[26] Yuan N. Prognostic value and association of sarcopenia for patients with bladder cancer undergoing transuretheral resection of bladder tumor. Master. Nanchang University; 2021.

[27] Almarzouq A, Kool R, Al Bulushi Y, Marcq G, Souhami L, Cury FL, et al. Impact of sarcopenia on outcomes of patients treated with trimodal therapy for muscle invasive bladder cancer. Urologic oncology 2022, 40(5):194.e115–194.e122. 10.1016/j.urolonc.2021.11.002 34862117

[28] Lin TY, Chen YF, Wu WT, Han DS, Tsai IC, Chang KV, et al. Impact of sarcopenia on the prognosis and treatment of lung cancer: an umbrella review. Discover Oncology 2022, 13(1):115. 36307591 10.1007/s12672-022-00576-0 PMC9616989

[29] Kim SH, Sin DS, Lim JY. Newly Diagnosed Sarcopenia and Alzheimer’s Disease in an Older Patient With Chronic Inflammation. Annals of geriatric medicine and research 2019, 23(1):38–41. doi: 10.4235/agmr.19.0005 32743285 PMC7387604

[30] Yoo JI, Ha YC, Choi H, Kim KH, Lee YK, Koo KH, et al. Malnutrition and chronic inflammation as risk factors for sarcopenia in elderly patients with hip fracture. Asia Pacific journal of clinical nutrition 2018, 27(3):527–532. doi: 10.6133/apjcn.082017.02 29737798

[31] Veasey-Rodrigues H, Parsons HA, Janku F, Naing A, Wheler JJ, Tsimberidou AM, et al. A pilot study of temsirolimus and body composition. Journal of cachexia, sarcopenia and muscle 2013, 4(4):259–265. doi: 10.1007/s13539-013-0113-y 23893509 PMC3830004

[32] Engelmann SU, Pickl C, Haas M, Kaelble S, Hartmann V, Firsching M, et al. Body Composition of Patients Undergoing Radical Cystectomy for Bladder Cancer: Sarcopenia, Low Psoas Muscle Index, and Myosteatosis Are Independent Risk Factors for Mortality. Cancers (Basel). 2023 Mar 15;15(6):1778. 36980664 10.3390/cancers15061778 PMC10046300

[33] Fukushima H, Koga F. Impact of sarcopenia in bladder preservation therapy for muscle-invasive bladder cancer patients: a narrative review. Transl Androl Urol. 2022 Oct;11(10):1433–1441. doi: 10.21037/tau-22-355 36386266 PMC9641057

[34] Zhu Y, Guo X, Zhang Q, Yang Y. Prognostic value of sarcopenia in patients with rectal cancer: A meta-analysis. PLoS One 2022, 17(6):e0270332. 35749415 10.1371/journal.pone.0270332 PMC9231737

[35] March C, Omari J, Thormann M, Pech M, Wienke A, Surov A. Prevalence and role of low skeletal muscle mass (LSMM) in hepatocellular carcinoma. A systematic review and meta-analysis. Clinical nutrition ESPEN 2022, 49:103–113. doi: 10.1016/j.clnesp.2022.04.009 35623801

[36] Jogiat UM, Sasewich H, Turner SR, Baracos V, Eurich DT, Filafilo H, et al. Sarcopenia Determined by Skeletal Muscle Index Predicts Overall Survival, Disease-free Survival, and Postoperative Complications in Resectable Esophageal Cancer: A Systematic Review and Meta-analysis. Annals of surgery 2022, 276(5):e311–e318. 35794004 10.1097/SLA.0000000000005452

[37] Jogiat UM, Bédard ELR, Sasewich H, Turner SR, Eurich DT, Filafilo H, et al. Sarcopenia reduces overall survival in unresectable oesophageal cancer: a systematic review and meta-analysis. Journal of cachexia, sarcopenia and muscle 2022. 10.1002/jcsm.13082 PMC9745498 36151845

[38] Zhang B, Yao W. Prognostic role of the systemic immune-inflammation index in biliary tract cancers: a meta-analysis of 3,515 patients. World J Surg Oncol 2022, 20(1):320. 36171621 10.1186/s12957-022-02783-z PMC9519406

[39] Li X, Zhang S, Lu J, Li C, Li N. The prognostic value of systemic immune-inflammation index in surgical esophageal cancer patients: An updated meta-analysis. Frontiers in surgery 2022, 9:922595. 36090319 10.3389/fsurg.2022.922595 PMC9459851

[40] Li D, Zhao X, Pi X, Wang K, Song D. Systemic immune-inflammation index and the survival of hepatocellular carcinoma patients after transarterial chemoembolization: a meta-analysis. Clinical and experimental medicine 2022. 10.1007/s10238-022-00889-y 36287310

